# TM-Doped B_12_N_12_ Nanocage (TM = Fe, Co, and Ni) As a Sensitive and Selective CO Gas Sensor: A Theoretical Study

**DOI:** 10.1021/acsomega.6c02910

**Published:** 2026-06-26

**Authors:** Adilson Luís Pereira Silva, Natanael de Sousa Sousa, Auro Atsushi Tanaka, Daniel da Silva de Sousa, Albérico Borges Ferreira da Silva

**Affiliations:** † Universidade Estadual do Maranhão, São Luís, MA 65055-310, Brazil; ‡ Universidade Federal do Maranhão, São Luís, MA 65080-805, Brazil; § Instituto de Química de São Carlos, Universidade São Paulo, CP 780, São Carlos, SP 13560-970, Brazil

## Abstract

The development of efficient sensors for hazardous gases remains a critical challenge, driving intense interest within the scientific community. In this theoretical investigation, the interactions between carbon monoxide (CO) and pristine B_12_N_12_, as well as transition-metal-doped nanocages (FeB_11_N_12_, CoB_11_N_12_, and NiB_11_N_12_), were examined using DFT at the B97*D*/6-31G­(d,p) level. The results reveal that CO is weakly physisorbed on the pristine B_12_N_12_ nanocage, indicating limited sensing capability. In contrast, metal doping markedly enhances adsorption strength and sensing performance, with the NiB_11_N_12_ system standing out for its moderate adsorption energy (*E*_ads_ = −0.76 eV) and high electronic sensitivity (80.9%). Beyond adsorption strength, selectivity coefficient analysis combined with molecular dynamics simulations demonstrates that NiB_11_N_12_ can effectively discriminate CO from common interfering gases, including CO_2_, N_2_O, CH_4_, and H_2_. Additionally, compared with other systems in the literature, NiB_11_N_12_ exhibits better sensitivity and selectivity, even under dry conditions or in very low humidity. Overall, this study advances the field by establishing NiB_11_N_12_ as a promising material for the selective detection of CO, thereby offering valuable insights for experimental fabrication and real-world applications.

## Graphical abstract

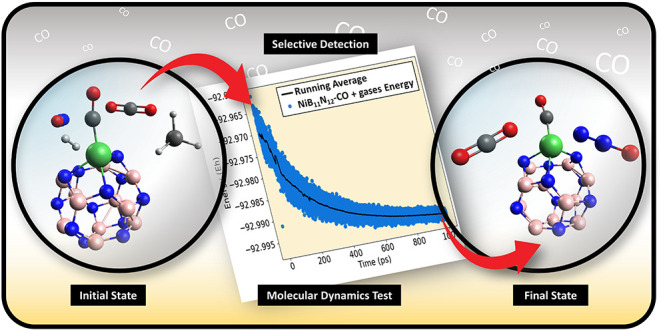

## 1 Introduction

Carbon monoxide (CO) is an extremely poisonous gas to humans, sometimes termed the “silent killer”. It is a colorless and odorless gas, known as one of the most toxic in the world, which disperses easily into confined spaces. Its high toxicity to humans is explained as a function of the preferred capacity of CO to bind strongly to the iron of hemoglobin (Hb), producing the complex carboxyhemoglobin (CO-Hb), causing headaches, dizziness, loss of consciousness, and, in more severe cases, death.^1,2^ Due to growing concerns regarding the health and safety risks associated with CO emissions. It is therefore essential to study and develop sensors for the detection of CO, which could be necessary. Its detection and control in residential and industrial environments are necessary to avoid potentially severe health problems in humans.

The development of boron nitride fullerene for hazardous gas sensor applications has attracted intense interest from the scientific community over the past few years due to its potential for fast response, high sensitivity, and high selectivity.^3–6^ Jensen and Toftlund were the first to show the possible structure for B_12_N_12_ nanocage.^7^ Other theoretical studies have indicated that the B_12_N_12_ nanocage was the most stable structure.^8,9^ However, Oku et al.^10^ synthesized and isolated the B_12_N_12_ nanocage for the first time, confirming that the structure obtained is consistent with the results of previous theoretical research. Moreover, significant efforts have been made to synthesize boron nitride nanocages.^11–14^ Recently, theoretical studies have investigated the interaction of nanocage surfaces (pristine and transition-metal-modified) and CO,^15–18^ as well as other molecules,^19–26^ revealing their promising potential for sensor applications.

Regarding the detection of the toxic gas CO, other theoretical studies using DFT with different materials are being developed.^27–34^ Milon Roy and Ahmed^27^ showed that the Co-doped BN nanosheets can be used to detect CO gas. The results obtained by Badalkhani-Khamseh et al.^28^ indicate that the Al-doped boron nitride biphenylene (BNBP) nanosheet has superior performance for detecting CO gas. In a study on monolayer CuCl, Pervaiz et al.^29^ demonstrated that CO adsorption at various temperatures leads to significant changes in conductivity. Rouhani et al.^30^ showed that Ga-doped phagraphene is sensitive to the concentration of CO gas. Also, a short recovery time is predicted for CO desorption from the surface. The results obtained by Naseem et al.^31^ indicate that the C_6_N_8_ surface is a superb option for CO and CO_2_ gases. In a study using CuO and 2CuO-doped WTe_2_, Wang et al.^32^ showed that the addition of CuO and 2CuO effectively enhances the adsorption capacity of the substrate toward CO gas. Moreover, we conclude that the two modified substrates are also capable of detecting and distinguishing CO, C_2_H_2_, and CH_4_ gases well. Duan et al.,^33^ based on their findings, concluded that the Pd-modified g-C_3_N_4_ system could be used for the selective detection of CO gas. Kanaani et al.^34^ tested TM-doped Zn_12_S_12_ nanocages (TM = Sc–Cu) for their interaction with CO toxic gas. The authors concluded that V- and Ni-doped Zn_12_S_12_ nanocages can be used as chemical sensors for CO gas.

To the best of our knowledge, no studies have investigated how the iron, cobalt, and nickel doping of B_12_N_12_ on the adsorption of CO in B_12_N_12_ was modified. Although recent studies have highlighted the potential of doped B_12_N_12_ structures for the detection of CO, 5-fluorouracil, hydroxyurea, H_2_Se, CNCl, and NO,^18–20,24–26^ additional studies are necessary. Therefore, based on this brief survey, this study aimed to investigate the influence of how iron, cobalt, and nickel doping affect the interaction between CO toxic gas and the FeB_11_N_12_, CoB_11_N_12_, and NiB_11_N_12_ nanocages using DFT.

## 2 Computational Details

All the quantum chemical calculations in the present study were performed using Gaussian 09 ^35^ and were based on the B97D functional.^16,18,36–38^ The 6-31G­(d,p) basis set was used for all atoms. To obtain a more precise assessment of the adsorption energy, we have also investigated our structures by considering dispersion correction (D2). The modification with iron, cobalt, and nickel on B_12_N_12_ resulted in three optimized structures, Fe-, Co-, and Ni-doped (FeB_11_N_12_, CoB_11_N_12_, and NiB_11_N_12_). Vibrational frequency calculations were also performed to ensure that all stationary points correspond to minima on the potential energy surface.

To investigate the stability of the nanocage structures, the cohesive energy (*E*_coh_) was calculated as follows

1 $$E_{\mathrm{coh}}=\frac{1}{24}\left(E_{\mathrm{nanocage}}-xE_{B}-12E_{N}-yE_{\mathrm{TM}}\right)$$

where the *E*_nanocage_ indicates the total energy of the optimized nanocage (pure and doped with nickel); *E*_B_, *E*_N_, and *E*_TM_ are the energy of B, N, and TM (Fe or Co or Ni) atoms, respectively; *x* and *y* are the numbers of B and TM atoms, respectively; *y* = 0 is for pristine B_12_N_12_ and *y* = 1 is for the doped structure (FeB_11_N_12_, CoB_11_N_12_, and NiB_11_N_12_). The HOMO–LUMO gap value is defined as

2 $$E_{\mathrm{gap}}=E_{\mathrm{LUMO}}-E_{\mathrm{HOMO}}$$

where *E*_gap_ is the HOMO–LUMO gap, and *E*_LUMO_ and *E*_HOMO_ are the energies of the lowest unoccupied molecular orbital and the highest occupied molecular orbital, respectively. The electronic sensitivity (Δ*E*_gap_) value for the interaction between the CO molecule and B_12_N_12_ or TMB_11_N_12_ nanocages is calculated using the following equation

3 $${\Delta E}_{\mathrm{gap}}=\left[\frac{{(E}_{\mathrm{gap}\left(\mathrm{nanocage}-\mathrm{CO}\right)}-E_{\mathrm{gap}\left(\mathrm{nanocage}\right)})}{E_{\mathrm{gap}\left(\mathrm{nanocage}\right)}}\right]\times 100\%$$

where *E*_gap(nanocage–CO)_ is the gap of B_12_N_12_–CO or TMB_11_N_12_–CO complexes, and *E*_gap(nanocage)_ is the gap of pure B_12_N_12_ or TMB_11_N_12_ nanocages. The Multiwfn postprocessing program has been applied to plot the density of states (DOS).^39^

The adsorption energy value (*E*_ads_) for the interaction between the CO molecule and B_12_N_12_ or NiB_11_N_12_ nanocages can be estimated as

4 $$E_{\mathrm{ads}}=E_{\left(\mathrm{nanocage}-\mathrm{CO}\right)}-\left(E_{\left(\mathrm{nanocage}\right)}+E_{\left(\mathrm{CO}\right)}\right)+E_{\mathrm{BSSE}}$$

where *E*_(nanocage–CO)_ is the energy of CO-bonded B_12_N_12_ or TMB_11_N_12_ complex, *E*_(nanocage)_ is the energy of pure B_12_N_12_ or TMB_11_N_12_ nanocages, *E*_(CO)_ is the energy of the CO molecule, and *E*_BSSE_ is the basis set superposition error (BSSE). To study the electronic charge transfer, the NPA charge transfer (*Q*_CT_) is defined as the charge difference between a CO gas molecule adsorbed on the B_12_N_12_ or TMB_11_N_12_ surfaces and an isolated gas molecule, which can be obtained by the following equation

5 $$Q_{\mathrm{CT}}=Q_{\left(\mathrm{nanocage}-\mathrm{CO}\right)}-Q_{\left(\mathrm{CO}\right)}$$

where *Q*_(nanocage–CO)_ *a*nd *Q*_(CO)_ represent the charges of a gas molecule adsorbed on the B_12_N_12_ or TM-doped surfaces and an isolated CO gas molecule, respectively. Finally, for the most sensitive system, further calculations were conducted to assess the selectivity of the nanocage toward CO gas in comparison with CH_4_, H_2_, CO_2_, N_2_O, and H_2_O gases.

To further evaluate the thermodynamic stability of the adsorption system and to strengthen the analysis of the nanocage’s selectivity toward CO in the presence of interfering gases, the most sensitive configuration was examined under more realistic conditions through molecular dynamics (MD) simulations. A 1000 ps trajectory was generated at 298.15 K, using a time step of 2 fs, while the force calculations were performed with the GFN-2 Hamiltonian as implemented in the xTB software.^40^

The graphs in Figure 1 present a comparative benchmark of total energies (*E*) and electronic band gaps (*E*_gap_) for CO, B_12_N_12_, NiB_11_N_12_, and NiB_11_N_12_–CO, using different functionals and basis sets (B97D, B3LYP, HSE06, PBE0, 6-31G­(d,p), def2-TZVPP, and LanL2DZ) including the perturbative MP2 method (see Table S1). It is observed that the B97D/6-31G­(d,p) functional provides consistent energies that are close to those obtained with larger basis sets, such as def2-TZVPP, indicating a good balance between computational cost and accuracy. For the band gap, B97D reproduces trends similar to B3LYP and PBE0, maintaining consistency in describing electronic modifications after doping and adsorption. HSE06 shows intermediate values, while MP2 overestimates the band gaps, as expected. The LanL2DZ basis set exhibits greater energetic discrepancy compared with other basis sets. Thus, B97*D*/6-31G­(d,p) demonstrates robustness and efficiency for describing BN-based systems containing transition metals and adsorption interactions.

**Figure 1:**
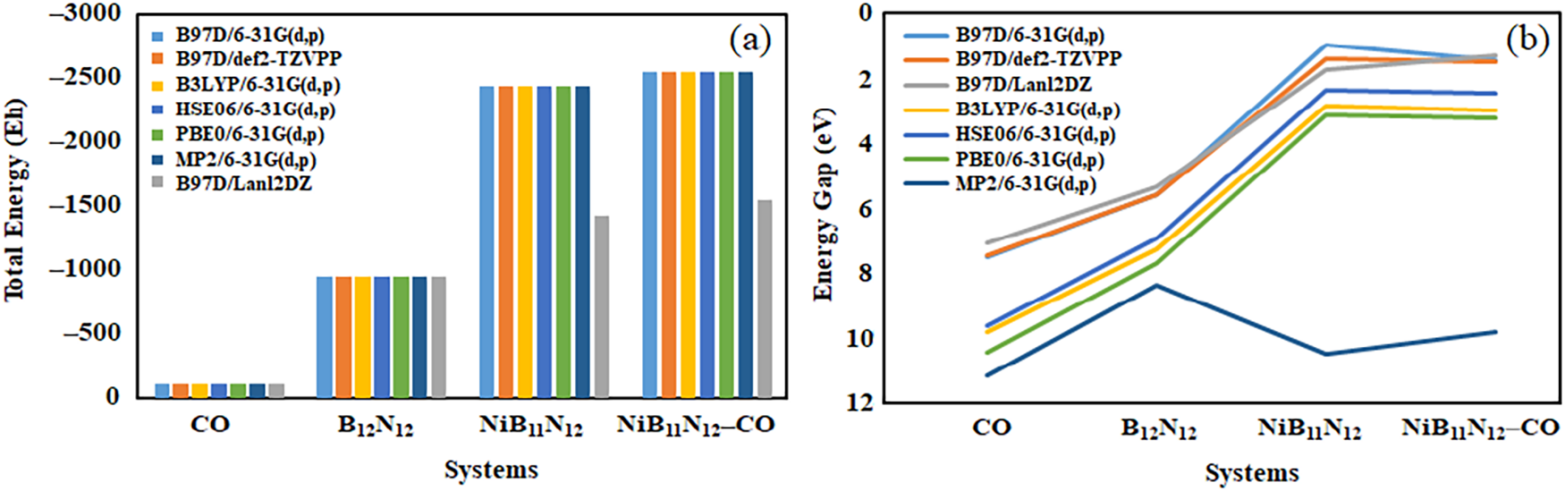
Total energy (a) and energy gap (b) of the CO, B_12_N_12_, NiB_11_N_12_, and NiB_11_N_12_–CO systems using B97D, B3LYP, HSE06, and PBE0 functionals, MP2 method, and 6-31G­(d,p), def2-TZVPP, and LanL2DZ basis set.

## 3 Results and Discussion

### 3.1 Structural and Electronic Properties of Pristine B_12_N_12_ and TMB_11_N_12_

The optimized structures of pure B_12_N_12_, FeB_11_N_12_, CoB_11_N_12_, and NiB_11_N_12_ nanocages are presented in Figure 2. For pristine B_12_N_12_, Figure 2a, the B–N bond lengths between two hexagonal rings (*b*_66_ = 1.449 Å) and between a hexagonal and a tetragonal ring (*b*_64_ = 1.494 Å), and the N–B–N bond angles of the hexagonal ring (θ_hex_ = 126.00°) and tetragonal ring (θ_tetra_ = 98.40°), which is consistent with previous studies.^18^ In Figure 2b–d, it is shown that when one iron, cobalt, or nickel atom is replaced by one boron atom, the local structural deformation of pure B_12_N_12_ nanocage occurs. This was evidenced by an increase in bond lengths of approximately 0.40 Å and a decrease in bond angles of approximately 30°. Furthermore, it was observed that the Fe, Co, and Ni atoms were pulled out of the nanocage surface after structural optimization due to the larger radius of the Fe, Co, and Ni atoms compared with the B atom.

**Figure 2:**
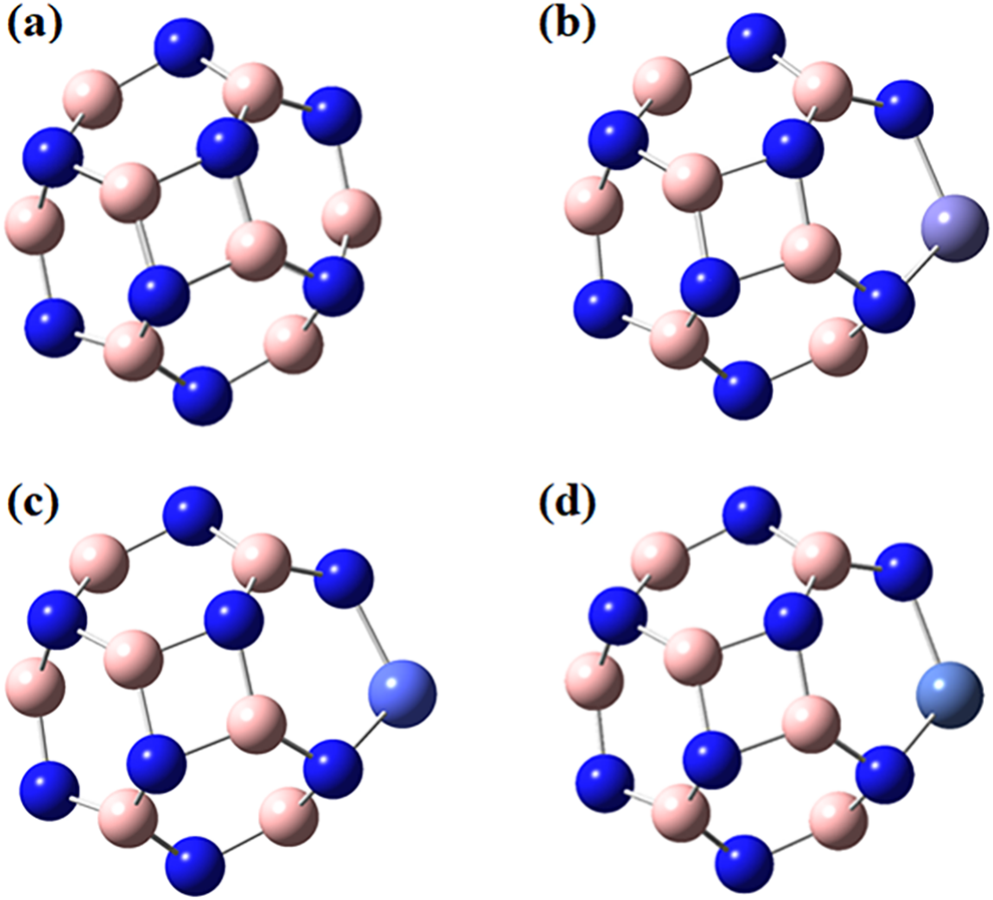
Optimized structures for isolated B_12_N_12_ (a), FeB_11_N_12_ (b), CoB_11_N_12_ (c), and NiB_11_N_12_ (d) nanocages. The figure represents Fe, Co, Ni, B, and N atoms in magenta, blue, light blue, pink, and dark blue, respectively.

The energetic stability of our nanocages was calculated using cohesive energy (eq 1): the values are presented in Table 1. The *E*_coh_ values showed that the B_12_N_12_ nanocage is more stable than the TM-doped structures, but the FeB_11_N_12_, CoB_11_N_12_, and NiB_11_N_12_ structures are possible. The more negative the cohesive energy values, the greater the energetic stability, as previously demonstrated.^41,42^ Furthermore, it was shown that the B_12_N_12_ nanocage presented a zero electric dipole and *E*_gap_ = 5.20 eV.^10,15,16^ We observed that *E*_gap_ decreased drastically with metal doping and is found in the range of 0.51–1.33 eV. These results for the decrease in the *E*_gap_ are in accordance with the results obtained previously.^4,17–19^ However, an increase in the dipole moment was observed after metal doping, indicating greater charge partition in the doped systems.

**Table 1:** Calculated Values of Cohesive Energy (*E*_coh_), Dipole Moment (DM), HOMO Energy (*E*_H_), LUMO Energy (*E*_L_), and Band Gap (*E*_gap_) for the Isolated Systems

system	*E*_coh_/eV	DM/Debye	*E*_H_/eV	*E*_L_/eV	*E*_gap_/eV
B_12_N_12_	–5.75	0.00	–6.71	–1.51	5.20
FeB_11_N_12_	–5.56	2.16	–5.27^a^	–4.09^a^	1.18^a^
			–4.55^b^	–4.04^b^	0.51^b^
CoB_11_N_12_	–5.60	1.90	–5.21	–4.11	1.10
NiB_11_N_12_	–5.64	1.41	–5.54^a^	–4.96^a^	0.58^a^
			–5.90^b^	–4.57^b^	1.33^b^

a Spin-up (α).

b Spin-down (β).

### 3.2 Structural, Energetic, and Electronic Properties of Pristine B_12_N_12_ and TMB_11_N_12_ Nanocages upon CO Adsorption

The optimized structures of B_12_N_12_–CO and TMB_11_N_12_–CO complexes are shown in Figure 3. It was observed that the interaction occurred at the carbon atom of the CO molecule, in accordance with work reported using B_12_N_12_ ^17–20^ and Fe-, Co-, and Ni-doped systems.^27,43,44^ The *E*_ads_, DM, *d*_cage–CO_, *d*_C–O_, and υ_C–O_ values for B_12_N_12_ and NiB_11_N_12_ nanocages after interacting with carbon monoxide are summarized in Table 2. The adsorption energy indicates that the attachment between the CO molecule and pristine B_12_N_12_ is physisorption, characterized by a small *E*_ads_ (weak interaction), which may not be effective at differentiating interfering gases. That is, CO gas is not favorably adsorbed on pristine B_12_N_12_, as shown by the Δ*G*_ads_ positive value, indicating a nonspontaneous process. However, the adsorption performance of CO gas on the nanocage surface improved when one boron atom was replaced by an iron, cobalt, or nickel atom (chemical adsorption of CO and Δ*G*_ads_ < 0), possibly aiding in the differentiation of interfering gases. This improvement is evidenced by the increase in adsorption energy from −0.07 eV for B_12_N_12_ to −0.76 eV for NiB_11_N_12_, −0.98 eV for CoB_11_N_12_, and −1.14 eV for FeB_11_N_12_. Nevertheless, the *E*_ads_ value for the Ni-doped nanocage remains suitable,^45^ indicating that the interaction between NiB_11_N_12_ and CO gas is moderate.

**Figure 3:**
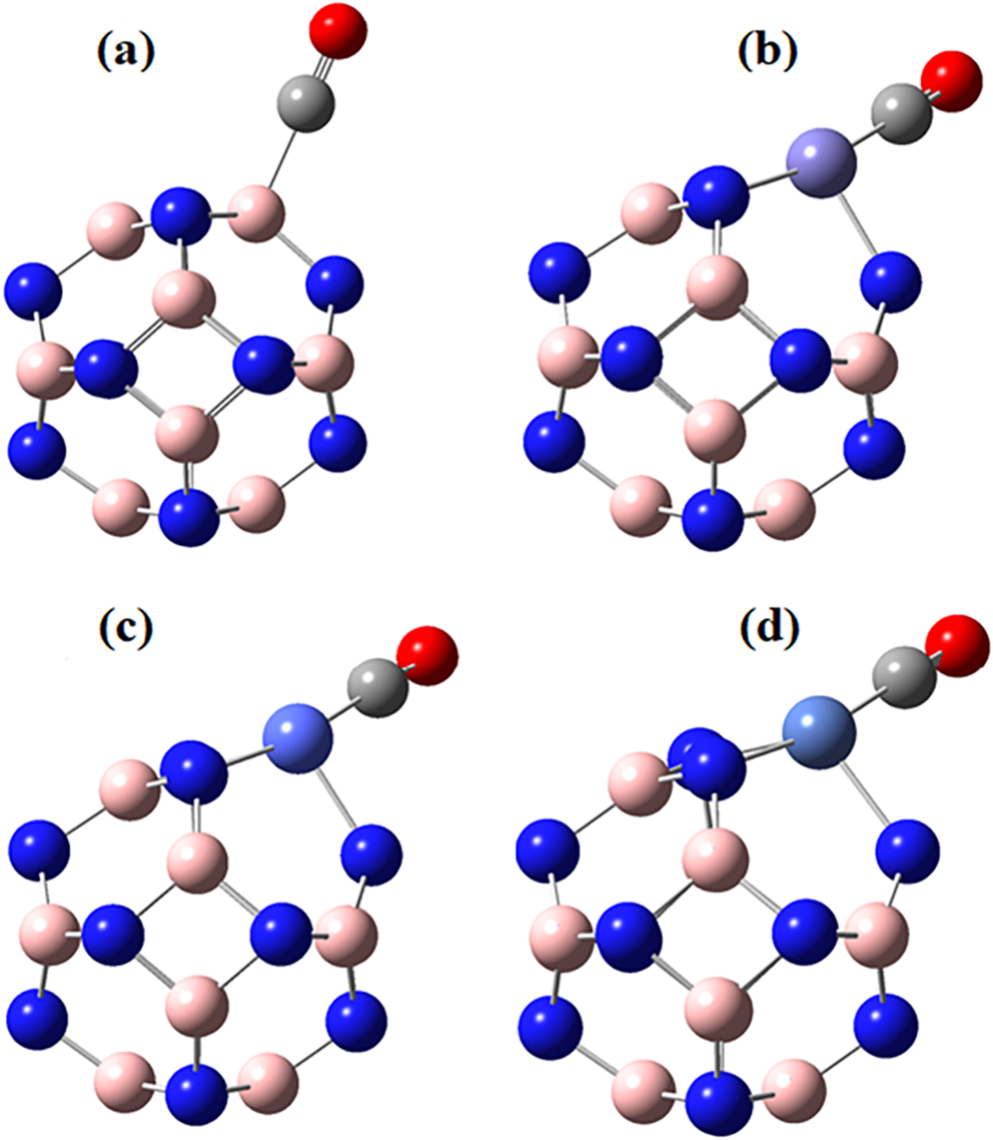
Optimized structures for complexes between carbon monoxide and isolated nanocages B_12_N_12_–CO (a), FeB_11_N_12_–CO (b), CoB_11_N_12_–CO (c), and NiB_11_N_12_–CO (d). The figure represents C, O, Fe, Co, Ni, B, and N atoms in gray, red, magenta, blue, light blue, pink, and dark blue, respectively.

**Table 2:** Calculated Values of Adsorption Energy (*E*_ads_), Dipole Moment (DM), Bond Length Cage–CO (*d*_cage–CO_), Bond Length C–O (*d*_C–O_), Bond Angle (θ), Stretching Frequencies C–O (*υ*_C–O_), Charge Transfer (*Q*_CT_), and Variation In Gibbs Free Energy (Δ*G*_ads_) for the Complexes Nanocage–CO (B_12_N_12_–CO, FeB_11_N_12_–CO, CoB_11_N_12_–CO, and NiB_11_N_12_–CO)

system	*E*_ads_/eV	DM/Debye	*d*_cage–CO_/Å	*d*_C–O_/Å	θ_(B/TM)–C–O_/°	υ_C–O_/cm^–1^	*Q*_CT_/|e|	Δ*G*_ads_/eV
B_12_N_12_–CO	–0.07	3.49	1.716	1.141	179.14	2155.91	+0.43	+0.19
FeB_11_N_12_–CO	–1.14	1.49	1.789	1.162	178.07	2024.05	–0.01	–1.15
CoB_11_N_12_–CO	–0.98	1.65	1.787	1.156	177.57	2061.14	+0.07	–1.00
NiB_11_N_12_–CO	–0.76	1.99	1.814	1.150	176.70	2101.78	+0.13	–0.77
CO		0.10		1.147		2124.61		

We also observed that there was a large increase in the dipole moment of B_12_N_12_ after the interaction with the CO molecule (from 0 to 3.49 D) and a small variation for the TM-doped nanocages after the interaction with CO gas; this behavior will directly reflect on the separation of charges after the interaction of the nanocages with CO. This interpretation is further supported by the analysis of the electrostatic potential map (MEP) and the electronic charge transferred between the nanocages and the CO molecule (*Q*_CT_) during adsorption. For the B_12_N_12_–CO complex, the region with the highest cationic character (depicted in blue) between the nanocage and the gas is more pronounced, with *Q*_CT_ = +0.43 |e|. In contrast, in the TMB_11_N_12_–CO complex (Figure 4b–d), this cationic region is less intense, with *Q*_CT_ values ranging from −0.01 to +0.13 |e|, revealing a smaller charge transfer and confirming the small variation in DM.

**Figure 4:**
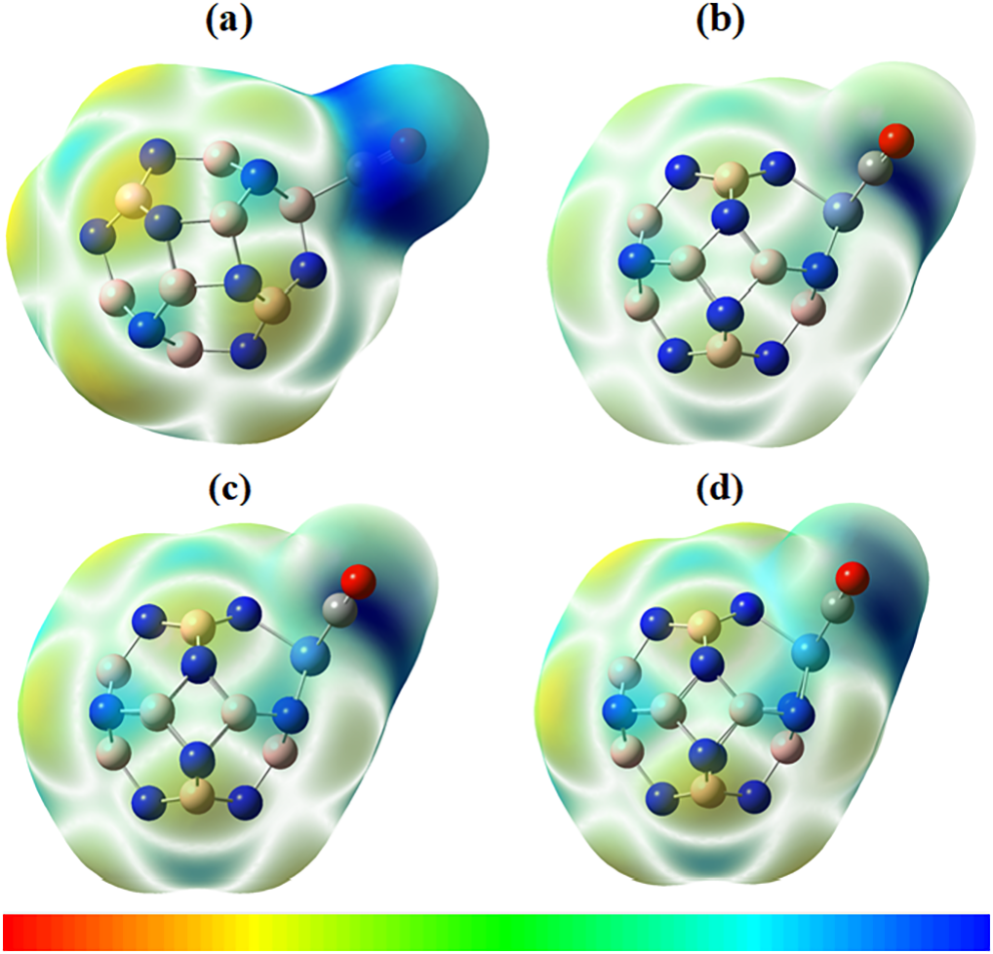
Molecular electrostatic potential for B_12_N_12_–CO (a), FeB_11_N_12_–CO (b), CoB_11_N_12_–CO (c), and NiB_11_N_12_–CO (d) complexes. Charge accumulation and depletion are represented by the red and blue regions, respectively.

The results revealed that there was an increase in the C–O bond length for the TM–B_11_N_12_–CO interaction, which can be explained due to the donation of electrons from occupied iron, cobalt, and nickel atomic orbitals to empty π* orbitals of carbon monoxide (back-bonding), while for the B_12_N_12_ nanocage there was a decrease in the C–O bond length compared to free CO, indicating that for this structure the effect of σ-donation is more effective than π-back-bonding. This can be explained using the NPA charge transfer to CO (negative charges or lower positive charges indicate a greater π-back-bonding contribution), and metals contribute to the formation of HOMO orbitals^26^ (the greater the contribution of the metal to the HOMO orbital, the greater the π-back-bonding, as shown for FeB_11_N_12_ (Figure 5b) and CoB_11_N_12_ (Figure 5c) nanocages.

**Figure 5:**
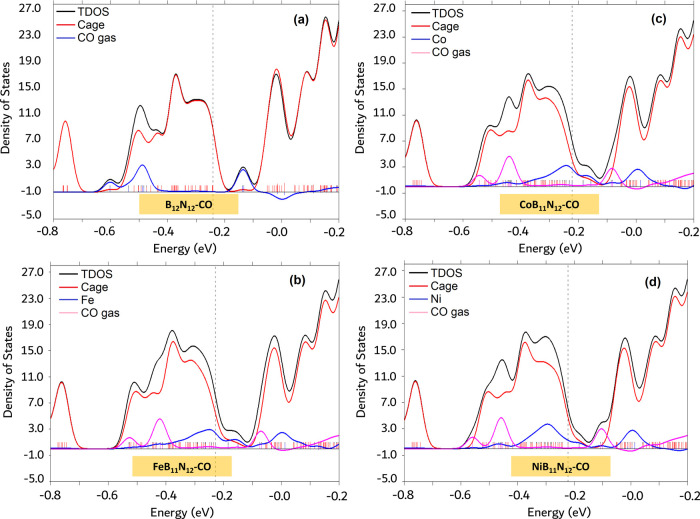
Total density of states (TDOS) and partial density of states (PDOS) diagrams of nanocages after interacting with carbon monoxide (B_12_N_12_–CO, FeB_11_N_12_–CO, CoB_11_N_12_–CO, and NiB_11_N_12_–CO).

Another approach for interpreting back-bonding involves analysis of υ_C–O_, as reported previously by us for iron complexes and modified nanocages with transition metals.^26,46^ The smaller υ_C–O_ indicates that a π-back-bonding takes place from the nanocages to CO. As shown in Table 2, the B_12_N_12_–CO complex υ_C–O_ value was higher than for noncoordinated carbon monoxide (lower *E*_ads_ and high σ-donation contribution). However, for the TMB_11_N_12_–CO complex, there was a decrease in υ_C–O_, indicating that for TM-doped, the interaction with CO was stronger. This interpretation is confirmed by the high *E*_ads_ observed, and the effect was more pronounced for the FeB_11_N_12_ nanocage. This can be explained using the HOMO (nanocage) and LUMO (CO) energies, which need to be closer. The HOMO values of B_12_N_12_ and FeB_11_N_12_ are about −6.71 and −4.55 eV, respectively. Thus, the energy difference between the LUMO of CO and HOMO of the pristine B_12_N_12_ is larger (Δ_gap_[HOMO­(B_12_N_12_–LUMO­(CO))] = 5.13 eV and υ_C–O_ = 2155.91 cm^–1^) compared to that of FeB_11_N_12_ (Δ_gap_[HOMO­(FeB_11_N_12_–LUMO­(CO))] = 2.97 eV and υ_C–O_ = 2024.05 cm^–1^), indicating that the π-back-bonding is more effective for the FeB_11_N_12_ nanocage. Nonetheless, we observed only for the NiB_11_N_12_ nanocage an intermediate value between σ-donation and π-back-bonding, which is an adequate value for applications in the detection of CO gas.

The *E*_H_, *E*_L,_ *E*_gap_, and Δ*E*_gap_ values for B_12_N_12_–CO and TMB_11_N_12_–CO systems studies after interaction with carbon monoxide are summarized in Table 3. Initially, we observed changes in the HOMO and LUMO energies, leading to changes in the *E*_gap_ values that are used to describe the electrical properties. A more detailed view of the changes in *E*_gap_ and the metals that contribute to the formation of HOMO and LUMO orbitals is obtained in Figure 5 (the values presented after interaction with the CO molecule). Despite the B_12_N_12_ nanocage exhibiting a high electronic sensitivity of 47%, as previously discussed, the *E*_ads_ values preclude its potential application as a sensor for CO detection. The FeB_11_N_12_ and CoB_11_N_12_ nanocages exhibit lower electronic sensitivity compared to B_12_N_12_ but high *E*_ads_ values, which limit their application to reversible CO detection. On the other hand, the DOS spectra and results presented in Table 3 revealed even more significant changes for the NiB_11_N_12_ nanocage, which showed the highest electronic sensitivity (80.9%), indicating that the interaction between NiB_11_N_12_ and CO gas was sensitive.

**Table 3:** Calculated Values of HOMO Energy (*E*_H_), LUMO Energy (*E*_L_), Band Gap (*E*_gap_), Electronic Sensitivity (Δ*E*_gap_), and Recovery Time (τ) for the Complexes between CO and Isolated Nanocages

system	*E*_H_/eV	*E*_L_/eV	*E*_gap_/eV	Δ*E*_gap_/%	τ/s
B_12_N_12_–CO	–6.32	–3.56	2.76	47.0	1.52 × 10^–13^
FeB_11_N_12_–CO	–5.99^a^	–4.80^a^	1.19^a^	0.84^a^	1.83 × 10^7^
	–5.46^b^	–4.86^b^	0.60^b^	17.6^b^	
CoB_11_N_12_–CO	–5.77	–4.77	1.00	9.09	3.63 × 10^4^
NiB_11_N_12_–CO	–5.84^a^	–4.80^a^	1.04^a^	80.9^a^	6.96
	–6.13^b^	–5.03^b^	1.10^b^	17.3^b^	
CO	–8.70	–1.58	7.12		

a Spin-up (α).

b Spin-down (β).

It is worth noting that the conductivity is an important factor to be considered in chemoresistive gas sensor applications. The conductivity (σ) of the sensing material before and after adsorbing gas molecules was evaluated using the equation^47^

6 $$\sigma =AT^{3/2}e^{{-E}_{\mathrm{gap}}/2k_{B}T}$$

where *A* (electron/m^3^ K^3/2^) is a constant, *T* is the thermodynamic temperature, *E*_gap_ is the HOMO–LUMO gap, and *k*_B_ is the Boltzmann constant. This equation shows that the electrical conductivity of the nanocages will exponentially change with a change in the *E*_gap_. This change can be converted to an electrical signal, which helps the detection of CO gas presence, as already shown experimentally.^48,49^ Finally, from the analysis of the change in *E*_gap_ results, it can be inferred that the NiB_11_N_12_ nanocage can be used in the development of sensors for the detection of CO gas.

To elucidate the electronic interactions associated with CO adsorption on the surfaces of the pristine and TM-doped B_12_N_12_ nanocages, charge density difference (CDD) calculations were performed, and the results are shown in Figure 6. The apparent charge redistribution occurs at the nanocage–CO interface, indicating strong coupling between the gas and substrate. Although the value of *E*_ads_ is physical in nature for the B_12_N_12_ nanocage, the predominance of van der Waals forces is expected. Unexpectedly, the CDD results indicate an effective interaction in the B_12_N_12_–CO system, corroborated by the significant NPA charge transfer observed after adsorption, with a pronounced σ-donation from CO to the B_12_N_12_ nanocage (*Q*_CT_ = +0.43 |e|). Furthermore, CO interacts more strongly with the metal-doped nanocages (TMB_11_N_12_), driven by a high π-back-donation contribution to FeB_11_N_12_ (*Q*_CT_ = −0.01 |e|) and CoB_11_N_12_ (*Q*_CT_ = +0.07 |e|) and a moderate π-back-donation to NiB_11_N_12_ (*Q*_CT_ = +0.13 |e|).

**Figure 6:**
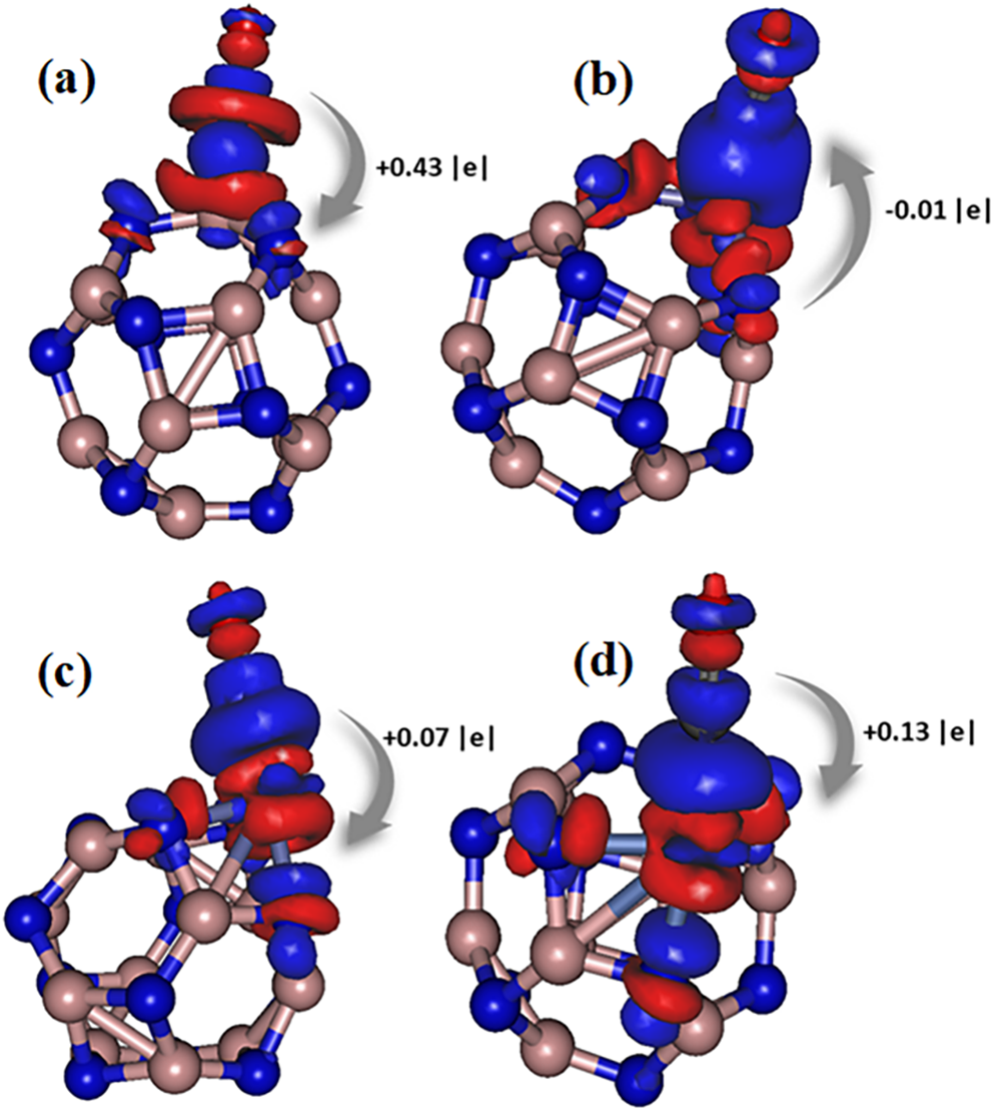
Charge density difference (CDD) of carbon monoxide gas on the pristine and TM-doped B_12_N_12_ nanocages with an isosurface of 0.001 e·Å^–3^ (B_12_N_12_–CO (a), FeB_11_N_12_–CO (b), CoB_11_N_12_–CO (c), and NiB_11_N_12_–CO (d)). Charge accumulation and depletion are represented by the red and blue regions, respectively. The arrows indicate the magnitude and direction of the electronic charge transfer.

Furthermore, to gain a deeper understanding of the nature of the new bonds formed between the boron, iron, cobalt, and nickel atoms of the nanocage and the carbon atom of the CO molecule, we analyzed the electron localization function (ELF),^50^ which is commonly used as a measure of electron pairing (localization). The ELF values range from zero, corresponding to complete electron delocalization (noncovalent bonding), to one, representing full electron localization (covalent bonding). From the analysis of Figure 7, it is possible to identify the formation of a predominantly covalent bond between the boron, iron, cobalt, and nickel atoms of the nanocage and the carbon atom of the CO molecule. This result is further supported by the adsorption energy (*E*_ads_ = −0.76 eV), which indicates a chemical interaction, and by the C–O vibrational frequency, which reveals a moderate effective π-back-donation for the NiB_11_N_12_ nanocage compared to high π-back-donation for the FeB_11_N_12_ and CoB_11_N_12_ nanocages. Otherwise, based on the *E*_ads_ values, noncovalent interaction–characteristic ELF features were initially expected for the interaction between B_12_N_12_ and CO. However, significant charge transfers from CO to the B_12_N_12_ nanocage, together with the CDD analysis, are consistent with the ELF results. Therefore, the physical or chemical nature of the interaction cannot be determined solely from *E*_ads_ values but must be correlated with complementary analyses such as *Q*_CT_, CDD, and ELF.

**Figure 7:**
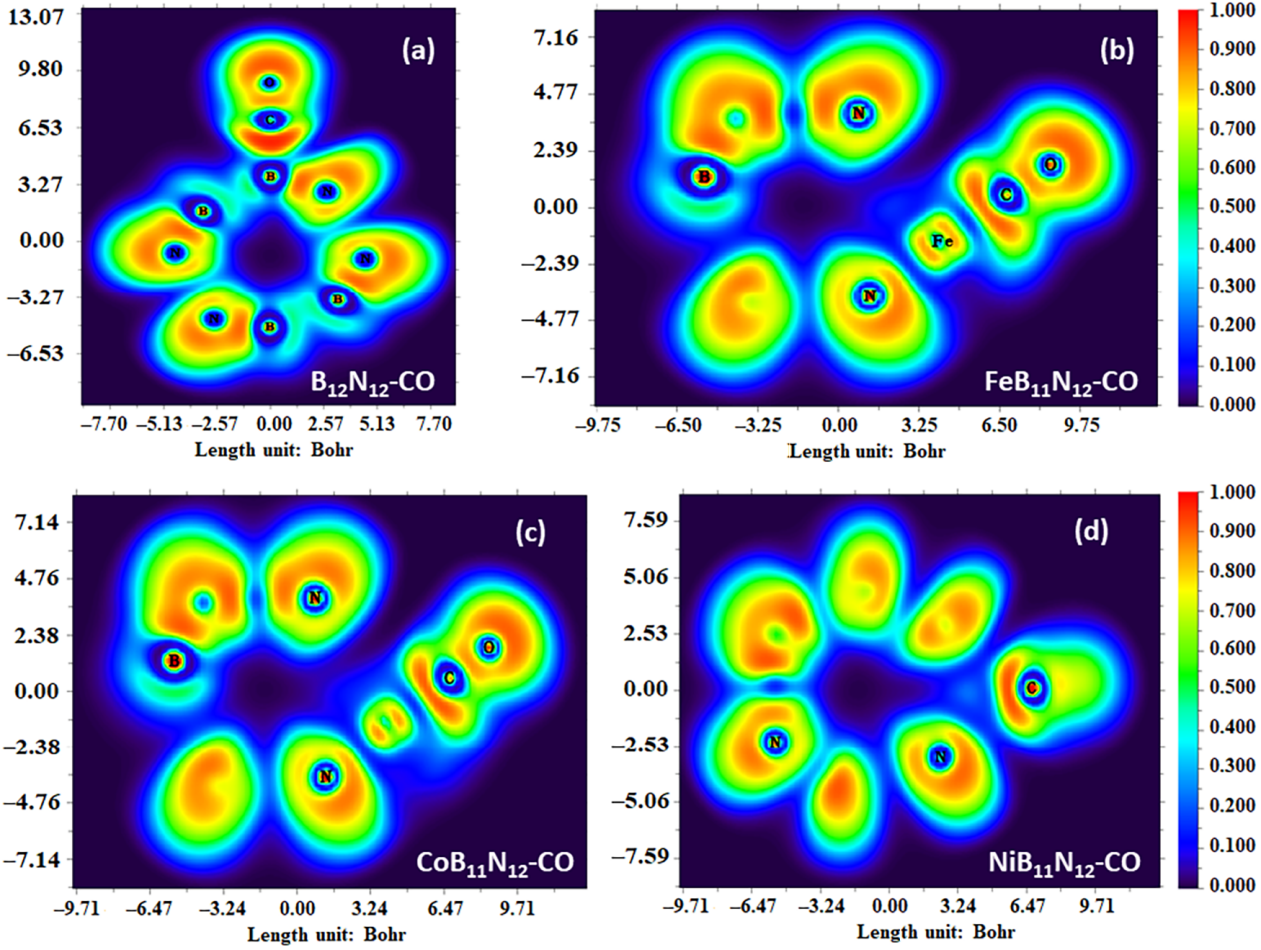
ELF plot for the B_12_N_12_–CO nanocage (a), FeB_11_N_12_–CO (b), CoB_11_N_12_–CO (c), and NiB_11_N_12_–CO complexes (d).

Another important parameter for determining the possibility of employing B_12_N_12_ and TMB_11_N_12_ nanocages for CO detection is the recovery time (τ), which is presented in Table 3. Although this parameter is experimentally obtained, in this study it was theoretically estimated using the transition state theory (van’t Hoff–Arrhenius), the following relationship between *E*_ads_ and τ^51^

7 $$\tau =v_{0}^{-1}e^{-E_{\mathrm{ads}}/k_{B}T}$$

where *v*_0_ is the attempt frequency (1 × 10^12^ s^–1^),^52,53^ *k*_B_ is the Boltzmann constant (8.62 × 10^–5^ eV K^–1^), and *T* is the thermodynamic temperature (298.15 K). It is worth noting that the attempt frequency can be calculated using transition state theory through the Eyring equation.^52^ In general, different attempt frequencies (or operating frequencies—UV, visible, and IR) can affect the response signal, the reversibility of detection, and the recovery rate.^53^ However, Kaewmaraya et al.^45^ caution that the binding energy employed in eq 7 should be interpreted carefully, as it is calculated based on the adsorption of a single molecule and therefore does not account for factors such as the gas concentration supplied to the sensor.

Based on eq 7, Peng et al.^51^ predicted that for an adequate adsorption energy range between −0.34 and −0.79 eV, the recovery time varies between 5 μs and 16 s, while an *E*_*ads*_ of −1 eV corresponds to a recovery time over 12 h. Kaewmaraya et al.^45^ showed that appropriate adsorption energy values should be between −0.3 eV < *E*_ads_ < −1.0 eV, and the recovery time should be in the range of a few seconds to a hundred seconds, which is acceptable for sensing applications. From these interpretations^50,54^ and the data presented in Tables 2 and 3, we can infer that a weak adsorption energy and low recovery time (*E*_ads_ = −0.07 eV and τ = 15.2 ps) indicate a limit for the selective detection of CO gas using B_12_N_12_ nanocages. However, for the interaction of CO with Fe- and Co-doped nanocages, high adsorption energies (greater than −0.98 eV) and very long recovery times (exceeding 10 h) are observed, indicative of the formation of strong chemical bonds, which limit their applicability as sensors. On the other hand, as the chemical interaction between CO and the NiB_11_N_12_ nanocage is moderate (*E*_ads_ = −0.76 eV and τ = 6.96 s), the material can achieve a reversible adsorption and desorption cycle in the application of actual gas sensing. Thus, from these data, the NiB_11_N_12_ nanocage is promising for applications such as a chemical sensor for carbon monoxide gas.

To gain a better understanding of the variation in recovery time, we employed three different attempt frequencies (10^12^ s^–1^—infrared, 5.2 × 10^14^ s^–1^—yellow light, and 10^16^ s^–1^—ultraviolet) at various temperatures ranging from 200 to 400 K.^53,55,56^ The calculated recovery times at 298.15 K were 6.96 s (infrared), 0.36 s (yellow light), and 0.70 ms (ultraviolet), revealing that the NiB_11_N_12_ nanocage can be effectively used for CO detection under all three light intensities tested. Furthermore, as shown in Figure 8, an increase in temperature leads to a decrease in τ, with the recovery time approaching zero at 358 K (infrared), 323 K (yellow light), and 265 K (ultraviolet). This indicates that, depending on the operating conditions, both light intensity and temperature can be modulated to achieve a sensor with a shorter recovery cycle, thus enhancing its potential for reuse.

**Figure 8:**
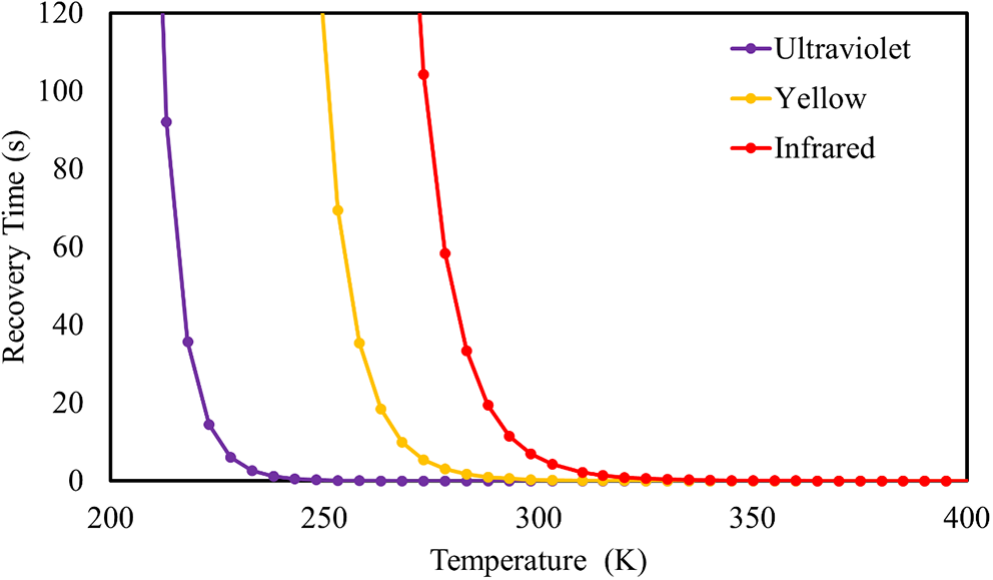
Variation in recovery time of the NiB_11_N_12_–CO adsorption system as a function of the temperature and attempt frequency.

Also, we have investigated the effect of the concentration of the CO molecules on the adsorption energy and the sensitivity of NiB_11_N_12_. To this aim, we have studied the adsorption of two and three CO molecules on the nanocage. The optimized structures of the complexes are shown in Figure 9. According to the results obtained by the adsorption of the second CO molecule, the interaction distance is slightly increased from 1.814 to 1.842 Å. In the adsorption of the third CO molecule, the interaction distance is drastically increased from 1.842 to 3.093 Å, indicating that this nanocage saturates by adsorbing up to two CO molecules on its surface. By increasing the number of CO molecules, the adsorption energies become less negative, from −0.76 eV (1 CO) to −0.45 eV (2 CO) and to −0.08 eV (3 CO), consistent with previous theoretical research.^54,57^ This may be due to the steric effect, as increasing the CO coverage around the nanocage, its tendency to accept another molecule is decreased. It is worth noting that the adsorption of the first three CO molecules occurs preferentially at the nickel site, in agreement with a previous study.^17^ The adsorption of a fourth molecule would only occur if the adsorption energy of the third molecule were favorable, which was not observed in this study.

**Figure 9:**
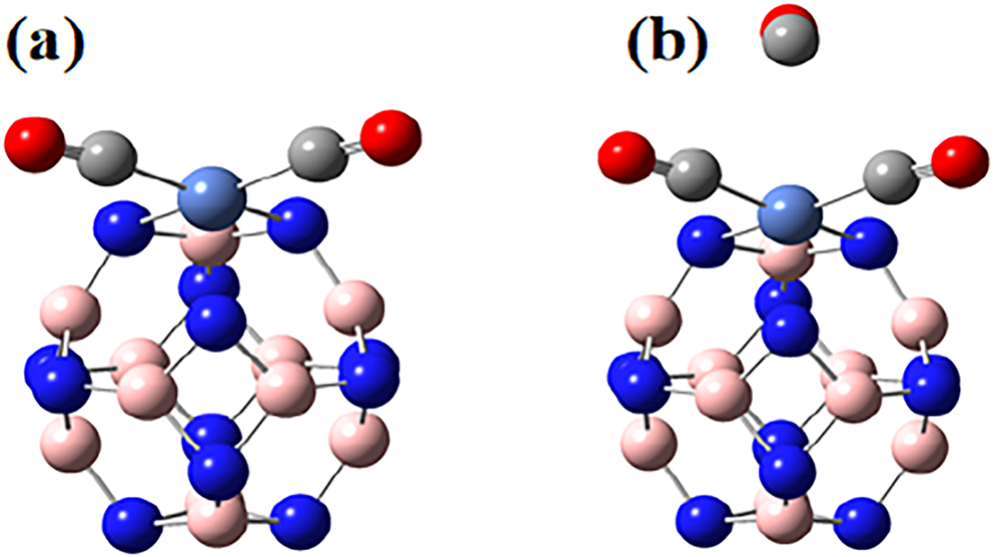
Optimized structures of complexes with two (a) and three (b) CO molecules adsorbed on NiB_11_N_12_. The figure shows C, O, Ni, B, and N atoms in gray, red, light blue, pink, and dark blue, respectively.

On the contrary, the decrease in adsorption energy (blue curve) did not result in a decrease in electronic sensitivity (red curve); in fact, there was a significant increase from 80.9% (1 CO) to 141.4 (2 CO) and then a slight decrease to 134.5% (3 CO) (see Figure 10), confirming that the electronic properties of the nanocage are much more affected by increasing the number of CO molecules and that the saturation of the system occurs with two CO molecules.

**Figure 10:**
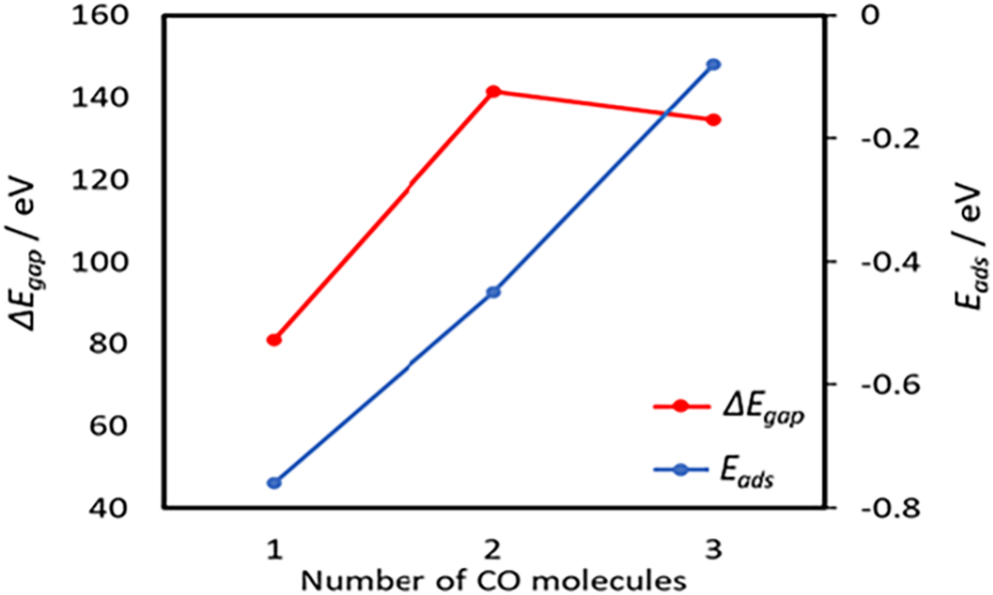
Correlation curve between the number of CO molecules adsorbed on the NiB_11_N_12_ nanocage. The blue curve represents the adsorption energy, and the red curve represents the electronic sensitivity.

In addition to electronic sensitivity, recovery time, and the effect of concentration, selective detection of carbon monoxide is a prime requirement for sensor applications. The selectivity of NiB_11_N_12_ to gases considered was evaluated by calculating the sensor response (*S*) and selectivity coefficient (κ) using the following equations^20,58^

8 $$S=\frac{\left|R_{\mathrm{gas}}-R_{\mathrm{pure}}\right|}{R_{\mathrm{pure}}}=\frac{\left|\frac{1}{\sigma_{\mathrm{gas}}}-\frac{1}{\sigma_{\mathrm{pure}}}\right|}{\frac{1}{\sigma_{\mathrm{pure}}}}=\frac{\left|\sigma_{\mathrm{pure}}-\sigma_{\mathrm{gas}}\right|}{\sigma_{\mathrm{gas}}}$$

9 $$\kappa_{\mathrm{CO}-\mathrm{int}}=\frac{S_{\mathrm{CO}}}{S_{\mathrm{int}}}$$

where σ_gas_ represents the conductivity of the NiB_11_N_12_–CO complex, σ_pure_ denotes the conductance of the isolated NiB_11_N_12_ nanocage, and *R* is the resistance. *S*_CO_ indicates the sensitivity to CO gas, and *S*_int_ indicates sensitivity to interfering gases commonly used in previous studies reported in the literature (CO_2_, N_2_O, H_2_, and CH_4_).^32,33,59–63^ However, κ_CO‑int_ represents the ratio of the sensitivity between CO and an interfering gas. In other words, selectivity refers to the ability of a gas sensor to detect a specific gas in a gas mixture.^64^ Furthermore, the optimized structures of NiB_11_N_12_–CO_2_, NiB_11_N_12_–N_2_O, NiB_11_N_12_–CH_4_, and NiB_11_N_12_–H_2_ complexes are shown in Figure 11.

**Figure 11:**
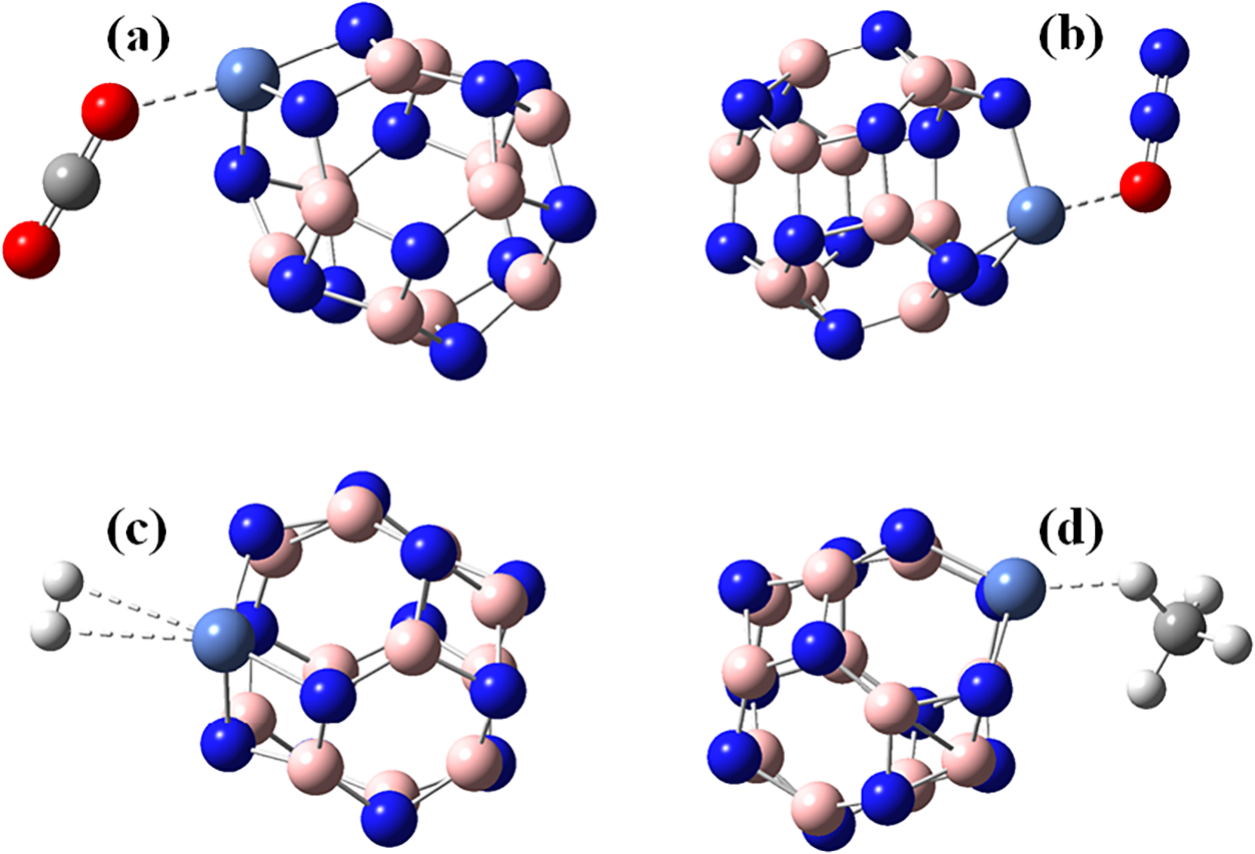
Optimized structures of NiB_11_N_12_–CO_2_ (a), NiB_11_N_12_–N_2_O (b), NiB_11_N_12_–H_2_ (c), and NiB_11_N_12_–CH_4_ (d) systems. The figure represents H, C, O, Ni, B, and N atoms in white, gray, red, light blue, pink, and dark blue, respectively.

The adsorption energy, sensor response, selectivity coefficient, charge transfer, and variation in Gibbs free energy are shown in Table 4, revealing that the NiB_11_N_12_ nanocage is more sensitive to carbon monoxide than the other studied gases. In addition, the NiB_11_N_12_–CO complex presented the highest *E*_ads_, *Q*_CT_, and ΔG_ads_ (more negative) compared with the same nanocage using interfering gases. Furthermore, as for selectivity, we observed that CO could be differentiated from all the studied interfering gases, and that the higher selectivity coefficient corresponds to greater differentiation between the gases.

**Table 4:** Calculated Values of Adsorption Energy (*E*_ads_), Electronic Sensitivity (Δ*E*_gap_), Sensitivity (*S*), Selectivity Coefficient (*κ*), Charge Transfer (*Q*_CT_), and Variation in Gibbs Free Energy (ΔG_ads_) for the Complexes between NiB_11_N_12_ Nanocage and CO, CO_2_, N_2_O, CH_4_, and H_2_ Gases

system	*E*_ads_/eV	Δ*E*_gap_/%	*S*	κ_CO‑int_	*Q*_CT_/|e|	Δ*G*_ads_/eV
NiB_11_N_12_–CO	–0.760	80.9	7701.97		0.13	–0.77
NiB_11_N_12_–CO_2_	–0.230	44.8	157.32	48.96	0.09	–0.03
NiB_11_N_12_–N_2_O	–0.190	44.3	148.46	51.88	0.10	–0.02
NiB_11_N_12_–CH_4_	–0.005	37.9	66.75	115.38	0.04	+0.13
NiB_11_N_12_–H_2_	–0.002	10.3	3.25	2369.84	0.02	+0.22

To investigate the thermodynamic stability of the studied adsorption system and to reinforce the analysis of the nanocage’s selectivity toward CO in the presence of interfering gases, the system was subjected to a 1000 ps molecular dynamics (MD) simulation at 298.15 K. The system consisted of a NiB_11_N_12_ nanocage surrounded by CO, CO_2_, N_2_O, CH_4_, and H_2_ gases. The graph in Figure 12 shows a gradual decrease in the system’s energy during MD simulation, which is related to the dispersion of CH_4_ and H_2_ gases, whereas CO_2_ and N_2_O molecules remain near the system but do not interact directly with the cage, as shown in Figure 12 (final state). In contrast, the CO molecule binds to the metal center via its carbon atom and stays there throughout the entire trajectory. The MD results corroborate both the thermodynamic stability and selectivity of the NiB_11_N_12_ nanocage toward CO gas, reinforcing its potential as a robust sensing material under practical operational conditions.

**Figure 12:**
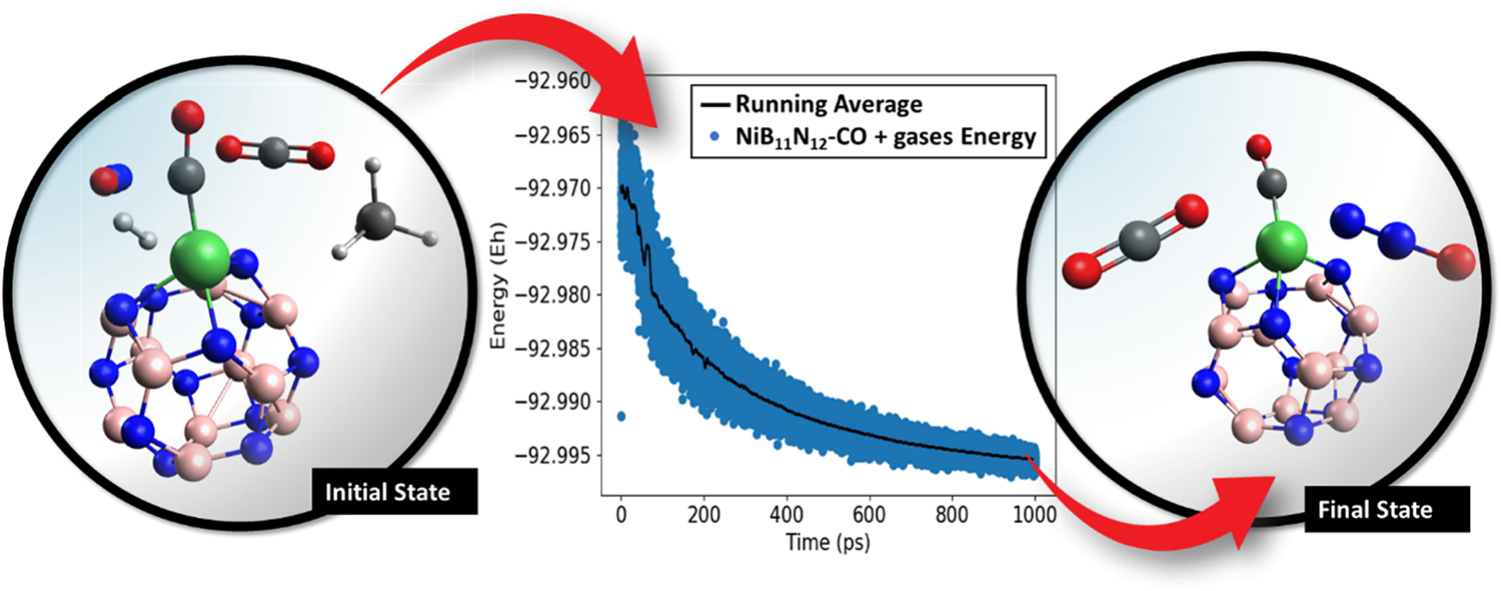
Molecular dynamics analysis of the NiB_11_N_12_ nanocage with CO, CO_2_, CH_4_, N_2_O, and H_2_ gases. Initial state (left side), trajectory graph, and final state of the trajectory (right side). The figure represents H, C, O, Ni, B, and N atoms in white, gray, red, green, pink, and dark blue, respectively.

UV–vis spectra and electronic transitions were estimated using TD-DFT calculations, with 100 roots and the same level of theory. The UV–vis spectra shown in Figure 13 reveal significant changes in the optical behavior of the NiB_11_N_12_ nanocage after CO adsorption. For the pristine nanocage, two main absorption bands are observed at 253 and 453 nm, indicating a typical response of systems with high symmetry and relatively uniform electronic distribution. After CO adsorption, the spectrum of the NiB_11_N_12_–CO complex exhibits a red shift of the absorption bands, with new maxima at 272, 375, and 530 nm. This shift, accompanied by a decrease in transition intensity, suggests a reduction in excitation energy and the formation of new electronic states induced by the interaction between CO orbitals and the Ni d orbitals. Therefore, the observed spectral modification confirms the occurrence of strong electronic coupling between the gas and the nanocage, reflecting changes in the electronic density, as shown before, and like the optical transitions of the material after adsorption.

**Figure 13:**
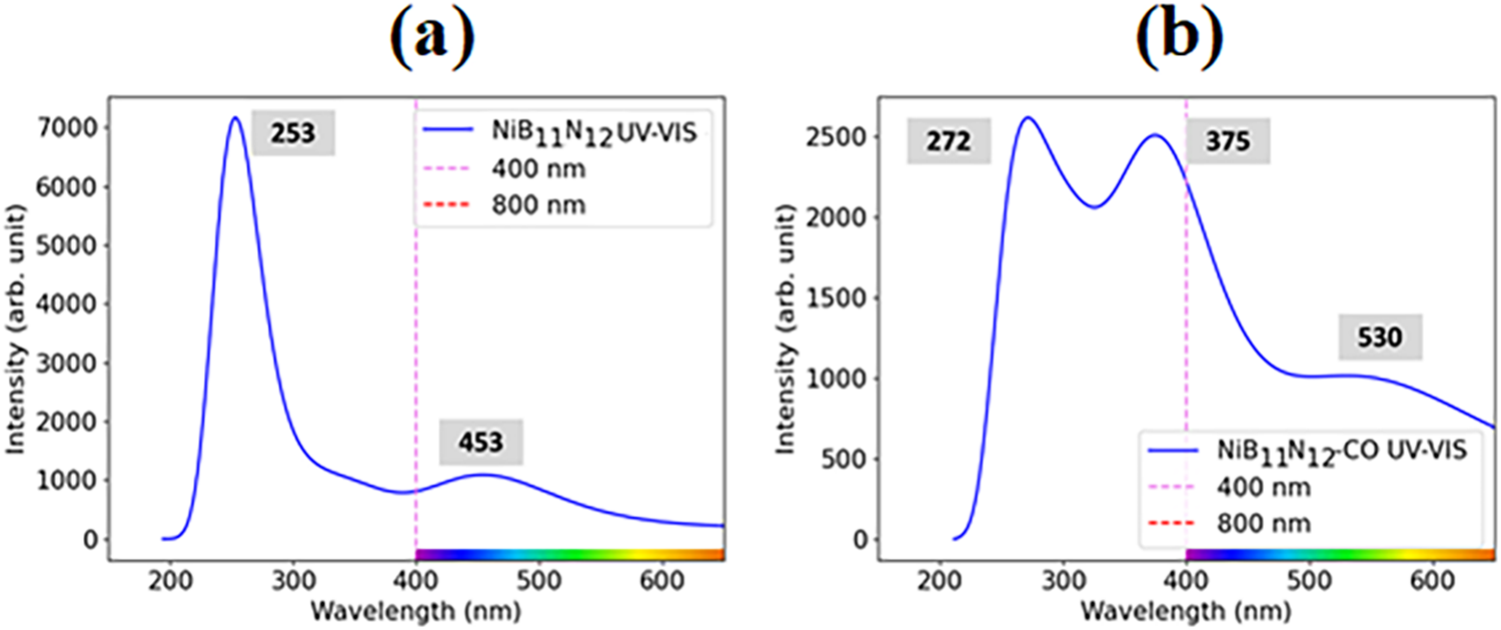
UV–vis spectra of the NiB_11_N_12_ (a) nanocage and NiB_11_N_12_–CO (b) complex.

The electronic transitions presented in Table 5 explain the origin of the bands observed in the UV–vis spectra of the NiB_11_N_12_ and NiB_11_N_12_–CO systems. For the pristine nanocage, the band at 253 nm (5.0 eV) arises from the H­(α) → L­(α) (92%) transition, characteristic of π→π* excitations localized on the BN rings, while the band at 453 nm (2.7 eV) involves H­(β) → L­(β) (64%) and H–1­(β) → L+1­(β) (34%) transitions with σ→π* character, reflecting the coupling between Ni and B–N framework orbitals. After CO adsorption, the NiB_11_N_12_–CO complex exhibits new bands at 272, 375, and 530 nm (red shift). The band at 272 nm (4.5 eV) reflects excitations localized on the cage, whereas the 375 nm (3.3 eV) band indicates coupling between Ni and CO orbitals. The low-energy band at 530 nm (2.2 eV), dominated by the H­(β) → L­(β) (72%) transition, corresponds to a metal-to-ligand charge-transfer process responsible for absorption in the visible region. These results confirm that CO adsorption significantly alters the electronic/optical properties of NiB_11_N_12_, highlighting its potential as a sensor.

**Table 5:** Calculated Electronic Transitions for NiB_11_N_12_ and NiB_11_N_12_–CO

system	λ_max_ (nm)	*f*	*E* (eV)	transitions
NiB_11_N_12_	0.034	253.2	5.0	H(α) → L(α) (92%)
	0.0085	453.2	2.7	H(β) → L(β) (64%)
				H–1(β) → L+1(β) (34%)
NiB_11_N_12_–CO	0.0095	271.9	4.5	H(β) → L(β) (27%)
				H–1(β) → L+1(β) (25%)
				H(α) → L(α) (18%)
				H–1(α) → L+1(α) (12%)
	0.015	375.1	3.3	H(β) → L(β) (35%)
				H(α) → L(α) (26%)
				H–1(β) → L+1(β) (18%)
	0.0055	530.0	2.2	H(β) → L(β) (72%)
				H(α) → L(α) (22%)

The last effect investigated in this study was how humidity influences the performance of CO detection, since it is already well-known that gas sensors are affected by humidity under real operating conditions. Therefore, understanding the effect of humidity on the sensing mechanism is of utmost importance. Li et al.^65^ clarified that, from a theoretical perspective, it is difficult to accurately represent the humidity percentages used in experiments, and that one possible way to model relative humidity is by interacting the nanocage with several water molecules. However, to investigate the effect of humidity, we adopted the strategy used by Li et al.^65^ and Jethawa and Chaudhari;^66^ that is, we sequentially interacted the system of interest (NiB_11_N_12_–CO) with one, two, and then three H_2_O molecules, as shown in Figure 14.

**Figure 14:**
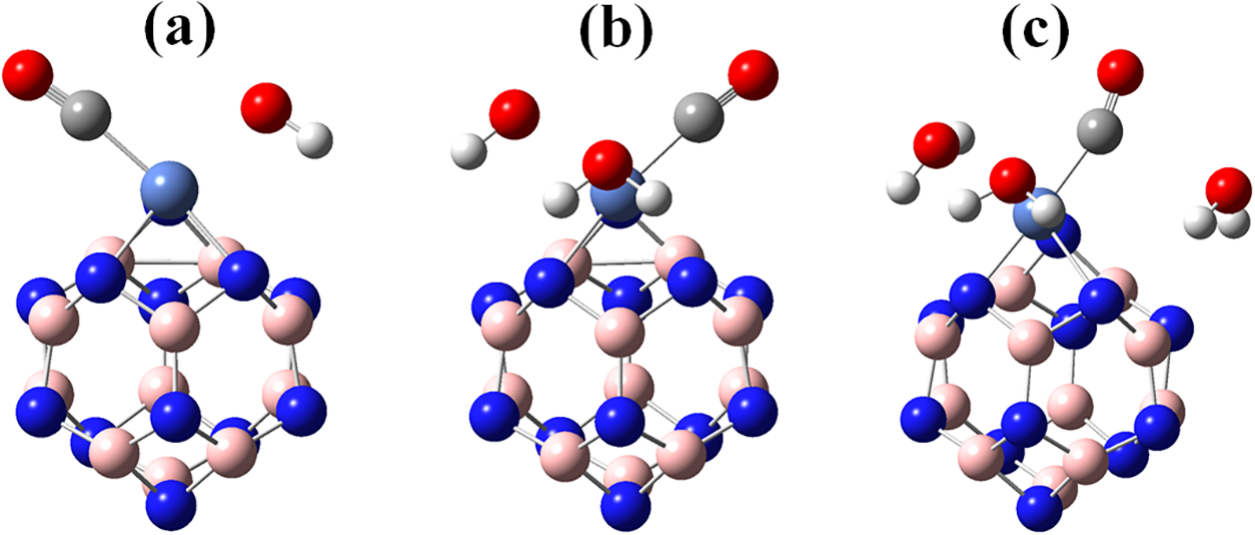
Optimized structures of the NiB_11_N_12_–CO complex in the presence of 1 H_2_O (a), 2 H_2_O (b), and 3 H_2_O (c) gas molecules. The figure represents H, C, O, Ni, B, and N atoms in white, gray, red, light blue, pink, and dark blue, respectively.

Before investigating the interaction of the NiB_11_N_12_–CO complex with water molecules, we first studied the interaction between the NiB_11_N_12_ nanocage and the H_2_O molecule to evaluate water adsorption. We found that the adsorption energy was approximately −0.75 eV, which is 0.01 eV lower than that calculated for CO adsorption, indicating a slight preference for CO over H_2_O.

Afterward, the CO and H_2_O molecules were positioned at the same distance from the nickel atom of the nanocage (1.81 Å), followed by geometry optimization. We observed an elongation of only 0.01 Å in the Ni–CO distance and 0.27 Å in the Ni–H_2_O distance (from 1.81 to 2.08 Å), indicating that after structural relaxation, the H_2_O molecule moved away from the nanocage (Figure 14a). In addition, the adsorption energy decreased from −0.76 eV (interaction of NiB_11_N_12_ with CO) to −0.49 eV (interaction of NiB_11_N_12_ with CO and one H_2_O molecule), indicating that water affects the adsorption process but not sufficiently to displace CO. Subsequently, upon addition of the second H_2_O molecule (Figure 14b), a greater separation of the water molecules from the nickel metallic center was observed (2.17 and 2.32 Å), with an adsorption CO energy of −0.16 eV. For the addition of the third H_2_O molecule (Figure 14c), an even larger separation of the water molecules from the nanocage was found (2.13, 2.37, and 3.44 Å), with an adsorption CO energy of +0.03 eV, indicating that an increase in humidity, while not displacing the CO gas, substantially affects CO adsorption. Therefore, these results demonstrate that CO detection using the NiB_11_N_12_ nanocage should preferably be performed under dry conditions or in very low humidity.

Finally, Table 6 summarizes the sensing performance of representative materials previously investigated for CO adsorption in theoretical studies.^15,27–34^ All reported values correspond to room-temperature conditions. Among the analyzed systems, the NiB_11_N_12_ nanocage exhibits a suitable recovery time (6.96 s) and the highest gas response (80.93%), outperforming most of the materials reported to date. Although the 2CuO–WTe_2_ system displays an electronic sensitivity comparable to that of NiB_11_N_12_, its relatively high adsorption energy (*E*_ads_ = −1.60 eV) may hinder sensor regeneration and long-term reusability.^18,45,58^ To mitigate this limitation, elevated operating temperatures have been proposed, with recovery times of approximately 1.8 s reported at 658 K.^32^ However, such conditions may restrict practical applications, particularly in low-power and portable sensing devices. In contrast, the balanced adsorption strength and favorable charge-transfer characteristics observed for NiB_11_N_12_ enable rapid signal recovery and high sensitivity under ambient conditions. These features suggest that this system provides a more advantageous compromise between responsiveness and reversibility compared to several previously reported materials.

**Table 6:** Comparison of Sensing Performance of the NiB_11_N_12_ Nanocage with Recent Theoretical Studies

sensing material	*E*_ads_/eV	Δ*E*_gap_/%	τ/s	other gases	references
B_12_N_12_	–0.43^a^	47.00	3.4 × 10^–5^	CO_2_, H_2_S, N_2_O, and SO_2_	Palomino-Asencio et al.^15^
Co-BNNS	–0.63^a^	20.42	9.58 × 10^–15^		Milon Roy and Ahmed^27^
Al-BNBP	–0.45^b^	16.10	1.1 × 10^–2^	CO_2_	Badalkhani-Khamseh et al.^28^
monolayer CuCl	–1.80^a^	14.21	4.70 × 10^10^	HF	Pervaiz et al.^29^
Ga-doped graphene	–0.30^b^	17.19			Rouhani et al.^30^
C_6_N_8_	–0.08^b^	0.26	4.06 × 10^–10^	CO_2_	Naseem et al.^31^
2CuO-WTe_2_	–1.60^a^	60.36	1.13 × 10^15^	C_2_H_2_ and CH_4_	Wang et al.^32^
Pd-*g*-C_3_N_4_	–2.34^a^	35.18	3.73 × 10^23^	C_2_H_2_ and C_2_H_4_	Duan et al.^33^
Ni-doped Zn_12_S_12_	–1.71^b^	33.35	4.41 × 10^16^		Kanaani et al.^34^
NiB_11_N_12_	–0.76^b^	80.93	6.96	CO_2_, N_2_O, CH_4_, H_2_, and H_2_O	this work

a Values without the BSSE correction.

b Values with BSSE corrections.

Therefore, the present results indicate that NiB_11_N_12_ is a promising candidate for the development of selective CO sensors operating at room temperature. Moreover, the theoretical trends identified in this work provide a solid framework for future experimental validation. Previous studies have demonstrated the feasibility of employing fullerene-based nanomaterials in gas-sensing platforms.^14,67,68^ In this context, the synthesis and electrochemical characterization of metal-modified B_12_N_12_ nanocages represent a relevant next step toward assessing their practical performance and technological applicability.

## 4 Conclusion

The interaction of CO gas with the pristine B_12_N_12_ and Fe-, Co-, and Ni-doped (FeB_11_N_12_, CoB_11_N_12_, and NiB_11_N_12_) nanocages was evaluated using DFT calculations at the B97D/6-31G­(d,p) level. The results indicate that CO gas is weakly physisorbed on pristine B_12_N_12_, whereas stronger chemisorption occurs on FeB_11_N_12_ and CoB_11_N_12_. Nevertheless, there is moderate CO adsorption (*E*_ads_ = −0.76 eV) on the surface of the NiB_11_N_12_ nanocage and an adequate recovery time of 6.96 s. Furthermore, the NiB_11_N_12_ nanocage showed a high electronic sensitivity of 80.9%. Moreover, the sensor performance analysis, based on the adsorption, sensitivity, selectivity, and molecular dynamics simulations, revealed that the NiB_11_N_12_ system is a promising candidate for carbon monoxide monitoring applications. In addition to being stable, it is also capable of distinguishing CO from the interfering gases studied (CO_2_, N_2_O, CH_4_, and H_2_), making it a promising selective sensor. It should be noted that the TD-DFT analysis revealed the NiB_11_N_12_ potential as an optical sensor. Thus, based on the results and compared to other systems reported in the literature, it highlights its potential for efficient CO gas detection under dry conditions at room temperature.

Finally, on the basis of the detailed theoretical evaluation of the structural, adsorptive, and electronic properties, we believe that, in addition to guiding a rational selection of promising candidates, it reduces experimental costs and directs the development of more efficient devices for the selective detection of CO. We also expect that these advances will strengthen the integration between computational and experimental research, aiming at the integration of the NiB_11_N_12_ nanocage into real sensing platforms, such as miniaturized devices operating under dry conditions rather than in humid atmospheres. However, the experimental synthesis of Ni-doped nanocages remains challenging. Therefore, further investigations, especially experimental validation and stability assessments, are essential to enable the practical application of this system in real-world sensing technology.

## Supplementary Material



## Notes

HOMO energy (*E*_H_), LUMO energy (*E*_L_), energy gap (*E*_gap_), and total energy (*E*_T_) of the NiB_11_N_12_ nanocage before and after CO adsorption calculated with B97D, B3LYP, HSE06, and PBE0 functionals and MP2 level (Table S1) (PDF)
