# Supplementary material for: TM-Doped B12N12 Nanocage (TM = Fe, Co, and Ni) As a Sensitive and Selective CO Gas Sensor: A Theoretical Study
Source: ACS Omega. 2026 Jun 26;11(28):42284–96. doi: 10.1021/acsomega.6c02910 (PMC13392892; doi:10.1021/acsomega.6c02910)
Supplement: Supplementary file 1 [file ao6c02910_si_001.pdf]

SUPPORTING INFORMATION

TM-doped $B_{12}N_{12}$ nanocage (TM = Fe, Co, and Ni) as a sensitive and selective CO gas sensor: a theoretical study

Adilson Luís Pereira Silva[1*], Natanael de Sousa Sousa[2], Auro Atsushi Tanaka[2], Daniel da Silva de Sousa[3], Albérico Borges Ferreira da Silva [3]

Affiliations

[1] *Universidade Estadual do Maranhão, 65055-310, São Luís, MA, Brazil.*
[2] *Universidade Federal do Maranhão, 65080-805, São Luís, MA, Brazil.*
[3] *Instituto de Química de São Carlos, Universidade São Paulo, CP 780, 13560-970, São Carlos, SP, Brazil.*
[*] Corresponding Author. E-mail address: adlpsilva@gmail.com

Table S1 - HOMO energy (E_H), LUMO energy (E_L), energy gap (E_{gap}), and total energy (E_T) of the $NiB_{11}N_{12}$ nanocage before and after CO adsorption calculated with B97D, B3LYP, HSE06, and PBE0 functionals and MP2 level.

Systems	Functional / basis set	E_H / eV	E_L / eV	E_{gap} / eV	E_T / eV
CO	B97D / 6-31G(d,p)	–8.70	–1.58	7.12	–113.24
$B_{12}N_{12}$		–6.71	–1.51	5.20	–955.43
$NiB_{11}N_{12}$		–5.54	–4.96	0.58	–2439.31
$NiB_{11}N_{12}$–CO		–5.84	–4.80	1.04	–2552.59
CO	B97D / def2-TZVPP	–9.11	–2.03	7.08	–113.29
$B_{12}N_{12}$		–6.91	–1.74	5.17	–955.74
$NiB_{11}N_{12}$		–6.19	–5.16	1.03	–2439.84
$NiB_{11}N_{12}$–CO		–6.05	–4.96	1.09	–2553.17
CO	B97D / Lanl2DZ	–8.84	–2.18	6.66	–113.21
$B_{12}N_{12}$		–7.21	–2.24	4.97	–955.17
$NiB_{11}N_{12}$		–9.02	–7.66	1.36	–1422.49
$NiB_{11}N_{12}$–CO		–8.43	–7.51	0.92	–1545.12
CO	B3LYP / 6-31G(d,p)	–10.13	–0.69	9.44	–113.31
$B_{12}N_{12}$		–7.75	–0.89	6.86	–956.14
$NiB_{11}N_{12}$		–7.17	–4.70	2.47	–2439.35
$NiB_{11}N_{12}$–CO		–6.96	–4.34	2.62	–2553.70
CO	HSE06 / 6-31G(d,p)	–10.01	–0.80	9.21	–113.19
$B_{12}N_{12}$		–7.67	–1.13	6.54	–955.13
$NiB_{11}N_{12}$		–7.05	–5.08	1.97	–2438.10
$NiB_{11}N_{12}$–CO		–6.84	–4.75	2.09	–2551.33
CO	PBE0 / 6-31G(d,p)	–10.43	–0.40	10.03	–113.18
$B_{12}N_{12}$		–8.08	–0.76	7.32	–955.06
$NiB_{11}N_{12}$		–7.45	–4.72	2.73	–2438.01
$NiB_{11}N_{12}$–CO		–7.23	–4.40	2.83	–2551.23
CO	MP2 / 6-31G(d,p)	–14.96	–4.19	10.77	–113.02
$B_{12}N_{12}$		–11.25	–3.28	7.97	–953.37
$NiB_{11}N_{12}$		–10.63	–0.52	10.11	–2435.39
$NiB_{11}N_{12}$–CO		–10.18	–0.75	9.43	–2548.44
